# NDM-1-producing *Enterobacter hormaechei* ST145 in Italy: genomic characterization of a multidrug-resistant high-risk clone

**DOI:** 10.1093/jacamr/dlag210

**Published:** 2026-09-18

**Authors:** A Piccirilli, G Alloggia, A Nicolucci, M Gambardella, M Di Domenico, C Cammà, L F Mincarelli, G Giordano, S Borriello, M Perilli

**Affiliations:** Department of Biotechnological and Applied Clinical Sciences, University of L’Aquila, L’Aquila, Italy; Department of Biotechnological and Applied Clinical Sciences, University of L’Aquila, L’Aquila, Italy; Department of Biotechnological and Applied Clinical Sciences, University of L’Aquila, L’Aquila, Italy; Infectious Diseases Unit, San Luca Hospital, Vallo della Lucania, Salerno, Italy; Molecular Biology and Genomic Unit, Istituto Zooprofilattico Sperimentale dell'Abruzzo e del Molise “G. Caporale”, Teramo, Italy; Molecular Biology and Genomic Unit, Istituto Zooprofilattico Sperimentale dell'Abruzzo e del Molise “G. Caporale”, Teramo, Italy; Molecular Biology and Genomic Unit, Istituto Zooprofilattico Sperimentale dell'Abruzzo e del Molise “G. Caporale”, Teramo, Italy; Clinical Pathology, San Luca Hospital, Vallo della Lucania, Salerno, Italy; Clinical Pathology, San Luca Hospital, Vallo della Lucania, Salerno, Italy; Department of Biotechnological and Applied Clinical Sciences, University of L’Aquila, L’Aquila, Italy

## Abstract

**Background and objectives:**

*Enterobacter hormaechei*, a member of the *Enterobacter cloacae* complex (ECC), is increasingly recognized as an opportunistic pathogen associated with multidrug resistance and nosocomial infections. The emergence of carbapenemase-producing ECC, particularly those harbouring *bla*_NDM-1_, represents a significant public health concern. This study aims to provide a comprehensive genomic characterization of an NDM-1-producing *E. hormaechei* strain (VDL/23) isolated from the bloodstream of a hospitalized patient in Italy.

**Methods:**

The isolate underwent hybrid WGS using Illumina short reads and Oxford Nanopore long reads. Genomic analyses were performed to characterize the resistome, virulome, plasmid content and associated mobile genetic elements. Conjugation assays were conducted using *Escherichia coli* K-12 as the recipient strain to assess plasmid transferability.

**Results:**

WGS identified the isolate as *E. hormaechei* subsp. *hoffmannii* ST145, a lineage sporadically reported worldwide but not previously described in Italy. The genome comprised a chromosome and three plasmids (pVDL/23-1, pVDL/23-2, pVDL/23-3). Chromosomal resistance included *bla*_ACT-90_, *fosA* and *oqxAB*, while the IncF-type plasmid pVDL/23-2 carried the main acquired resistance genes, including *bla*_NDM-1_, *bla*_CTX-M-15_, *bla*_OXA-1_ and additional determinants for aminoglycoside, trimethoprim and chloramphenicol resistance. Multiple insertion sequences and IncF replicons supported a mobile genetic context, and conjugation assays confirmed plasmid-mediated transfer of resistance.

**Conclusions:**

To the best of our knowledge, this study reports the first identification of an NDM-1-producing *E. hormaechei* ST145 isolate in Italy. The coexistence of extensive resistance determinants and mobile genetic elements highlights the emergence of a high-risk lineage and reinforces the need for genomic surveillance and infection control strategies to limit its dissemination in healthcare settings.

## Introduction

The genus *Enterobacter* comprises 22 species that have been reported as opportunistic pathogens in humans, animals and plants. Among them, *Enterobacter aerogenes*, *Enterobacter cloacae* and *Enterobacter hormaechei* are the species most frequently isolated from clinical infections.^1^ Owing to their high adaptability to antimicrobial agents, these bacteria commonly colonize immunocompromised patients and are associated with severe infections.^2^  *E. hormaechei* is a member of the *E. cloacae* complex (ECC) and is genetically closely related to other species within this group.^3^ This species is recognized for its genomic versatility and strong adaptive capacity in both clinical and environmental settings. Five subspecies of *E. hormaechei* have been identified: *E. hormaechei* subsp*. hormaechei*, *E. hormaechei* subsp. *hoffmannii*, *E. hormaechei* subsp. *oharae*, *E. hormaechei* subsp. *steigerwaltii* and *E. hormaechei* subsp. *xiangfangensis*.^4^ Clinical isolates of *E. hormaechei* are being increasingly identified in healthcare settings. At baseline, this species exhibits intrinsic resistance to penicillins and first- and second-generation cephalosporins. This natural resistance phenotype is driven by the production of a chromosomally encoded, inducible AmpC β-lactamase. Moreover, *E. hormaechei* frequently acquires plasmid-mediated extended-spectrum β-lactamases (ESBLs) and carbapenemases, including *bla*_KPC_, *bla*_NDM_ and *bla*_OXA-48_, which confer resistance to most β-lactam antibiotics.^5,6^ Recent genomic studies have shown that *E. hormaechei* harbours an open and highly dynamic pan-genome, shaped by horizontal gene transfer mediated by plasmids, integrative conjugative elements, prophages and transposons.^7^ This genomic plasticity facilitates the acquisition and dissemination of antimicrobial resistance and virulence determinants across diverse ecological niches, including hospital wastewater, food sources and the human gut.^8^ The epidemiology of carbapenem-resistant *Enterobacterales* (CRE) in Italy has been dominated by KPC-producing isolates. However, recent national surveillance data indicate a progressive increase in NDM-producing *Enterobacterales*, with the emergence of several outbreaks involving different bacterial species and sequence types (STs), in northern, central and southern Italy.^9–12^ Although *E. hormaechei* is increasingly recognized as an important reservoir of carbapenemase genes, reports involving ST145 remain limited. Nevertheless, recent genomic studies have identified ST145 among the predominant lineages associated with NDM-producing ECC isolates in France, suggesting its potential role in the dissemination of NDM-carrying mobile genetic elements (MGEs).^13^ In this context, we analysed the whole genome of an NDM-producing *E. hormaechei* strain isolated from the blood of a hospitalized patient.

## Methods

### Strain isolation and antimicrobial susceptibility

In 2023, an ECC strain (VDL/23) was isolated from blood culture of a hospitalized patient in S. Luca Hospital (Vallo della Lucania, Italy). Blood culture was processed using the BACTEC 9240 automated system (Becton Dickinson, USA). Species identification and antimicrobial susceptibility testing were performed using the VITEK 2 automated system (bioMérieux, Marcy-l'Étoile, France). Carbapenem resistance was further confirmed for imipenem and meropenem by Etest (bioMérieux, Marcy-l'Étoile, France). Minimum inhibitory concentrations (MICs) were determined by broth microdilution using a bacterial inoculum of 5 × 10^5^ CFU/mL, according to CLSI guidelines.^14^ The VDL/23 strain was subsequently identified as *E. hormaechei* via WGS.

### Whole-genome sequencing (WGS) analysis

WGS of the *E. hormaechei* VDL/23 strain was performed using a combination of short- and long-read sequencing technologies. Short-read libraries were prepared using the Illumina DNA Prep kit (Illumina Inc., San Diego, CA, USA) and sequenced on the Illumina MiSeq platform with a 2 × 300 bp approach. For long-read sequencing, libraries were prepared using the Native Barcoding Kit 24 V14 (SQK-NBD114.24) according to the manufacturer’s instructions and sequenced on the Oxford Nanopore Technologies (ONT) GridION benchtop device. Hybrid assembly was executed using Unicycler v0.5.1, and genome annotation was performed via Prokka v1.14.6 (https://github.com/tseemann/prokka). The whole-genome sequence of the VDL/23 strain has been deposited in the GenBank database under BioProject accession number PRJNA1463182. Species identification and sequence typing were carried out using the ribosomal multilocus sequence typing (rMLST) and MLST schemes available in the PubMLST database (https://pubmlst.org/). Subspecies assignment was confirmed by Average Nucleotide Identity (ANI) analysis using FastANI (>98% cut-off) against the *E. hormaechei* subsp. *hoffmannii* DSM 14563 reference genome (GenBank: GCA_050773135.1) (https://usegalaxy.eu/). Plasmid identification and mobility potential were characterized using MOB-suite v3.1.4. Antibiotic resistance genes (ARGs), MGEs and virulence factors were identified as previously reported.^15^ Plasmid maps were generated and visualized using SnapGene v8.2.2.

### Gene transfer assay

Conjugation experiments were performed using *E. hormaechei* VDL/23 as the donor strain and *E. coli* K-12 as the recipient strain. Transconjugants were selected on Luria-Bertani agar plates supplemented with both ceftazidime (32 mg/L) and meropenem (16 mg/L) in combination with streptomycin (250 mg/L). The detection limit of the assay was approximately 5 × 10^−7^ transconjugants per recipient cell.

## Results and discussion

**Figure 1.**
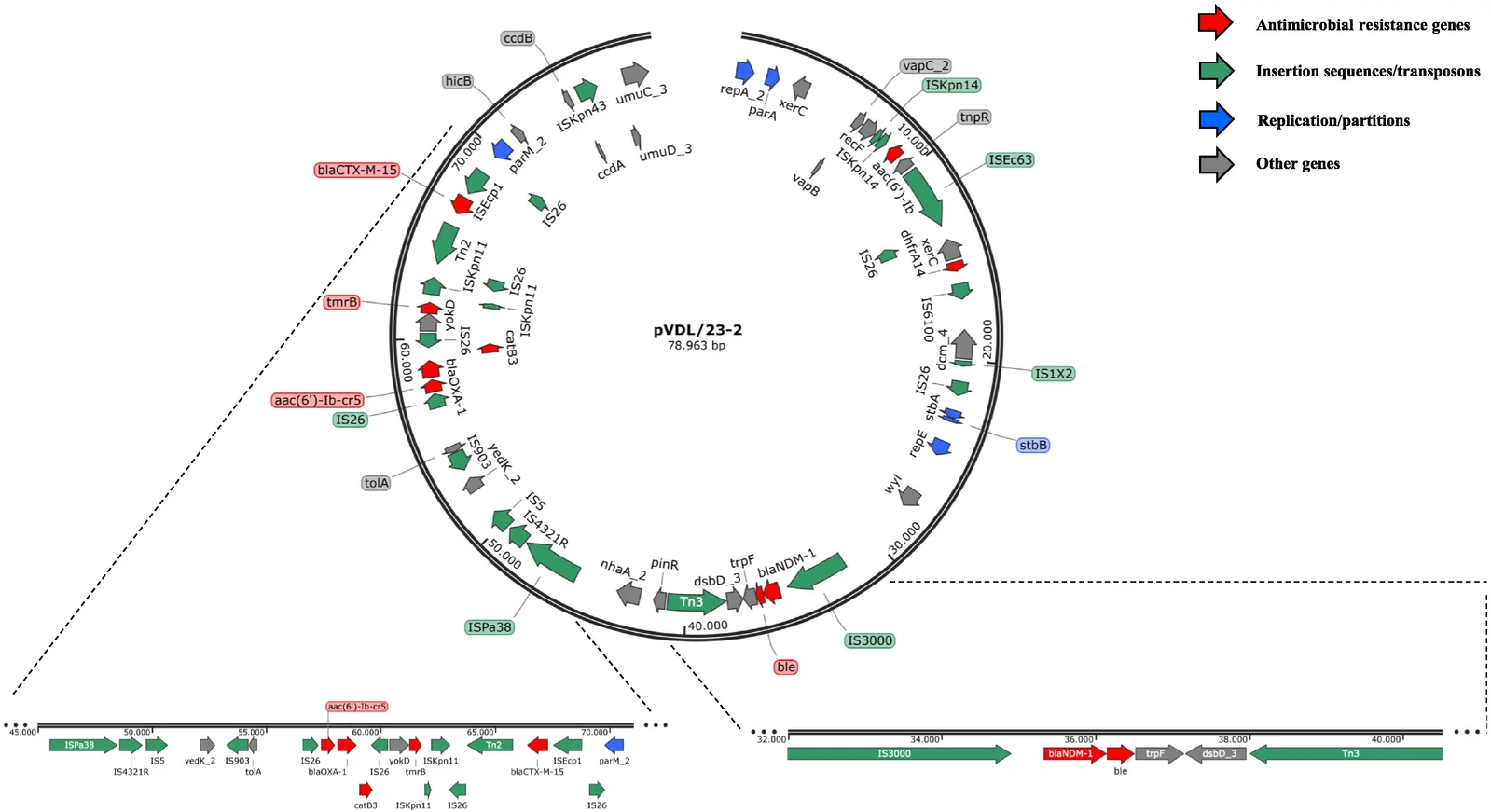
Circular map of plasmid pVDL/23-2 (78963 bp). Arrows indicate predicted open reading frames and transcriptional orientation. Gene functions are categorized into antimicrobial resistance genes, insertion sequences/transposons, replication/partition systems, and other genes, as detailed in the figure legend.

Antimicrobial susceptibility testing showed that the VDL/23 isolate was resistant to penicillins, cephalosporins, carbapenems, β-lactam/β-lactamase inhibitors and aminoglycosides, remaining susceptible only to colistin, ciprofloxacin and trimethoprim/sulfamethoxazole. The MICs for meropenem-vaborbactam, aztreonam and imipenem-relebactam were 1 mg/L, 48 mg/L and 3 mg/L, respectively. MLST identified the isolate as ST145, a lineage sporadically reported worldwide, although its occurrence remains limited compared with other high-risk STs.^16,17^ To our knowledge, no cases of ST145 have been described in Italy. FastANI-based analysis classified the isolate as *E. hormaechei* subsp. *hoffmannii*, consistent with recent reports indicating this subspecies as a predominant member of the ECC frequently associated with multidrug resistance (MDR) and nosocomial infections.^2^ WGS revealed a 4 679 906 bp chromosome with a GC content of 55.25%, along with three plasmids, hereafter referred to as pVDL/23-1 (157 232 bp), pVDL/23-2 (78 963 bp) and pVDL/23-3 (2494 bp). As shown in Table 1, the resistome was primarily distributed between the chromosome and plasmid pVDL/23-2. No ARGs were detected on plasmids pVDL/23-1 and pVDL/23-3. The chromosomal resistome includes genes conferring resistance to fosfomycin (*fosA*), fluoroquinolones (*oqxA*, *oqxB*) and β-lactams via the AmpC enzyme *bla*_ACT-90_. In contrast, pVDL/23-2, an IncFIA (HI1) plasmid, carried a dense cluster of acquired resistance determinants, including genes encoding resistance to aminoglycosides [*aac(3)-IIa*, *aac(6’)-Ib*], trimethoprim (*dfrA14*), chloramphenicol (*catB3*) and bleomycin (*ble*). Importantly, the plasmid also harboured *aac(6’)-Ib-cr5*, a well-recognized plasmid-mediated quinolone resistance (PMQR) determinant; alongside its aminoglycoside-modifying activity, this gene explicitly contributes to reduced fluoroquinolone susceptibility. Furthermore, the plasmid carried key β-lactamases *bla*_CTX-M-15_, *bla*_OXA-1_ and *bla*_NDM-1_. Genetic mapping of pVDL/23-2 identified two major resistance loci enriched in MGEs (Figure 1). The *bla*_NDM-1_ gene was located within a ∼10 kb region flanked upstream by IS*3000* and downstream by the *ble* gene and included a Tn3 family transposase, consistent with previously described NDM-associated platforms and indicative of mobilization potential. The presence of *bla*_NDM-1_ is of high clinical significance as it encodes a metallo-β-lactamase capable of hydrolysing nearly all β-lactam antibiotics, including carbapenems. Its localization on a plasmid facilitates horizontal dissemination, posing a substantial risk for the spread of high-level resistance.^18,19^ A second ∼25 kb region harboured *bla*_OXA-1_ and *bla*_CTX-M-15_, the latter positioned downstream of an IS*Ecp1* element, along with additional resistance genes such as *aac(6’)-Ib-cr5*, *aac(3)-IIa* and *catB3*, and multiple insertion sequences (IS*26*, IS*Kpn11*) and a Tn2 transposon, supporting a composite structure shaped by recombination events. The presence of these high-priority resistance determinants within such a plastic and mobile genetic environment underscores the significant clinical risk and the potential for horizontal gene transfer associated with the *E. hormaechei* VDL/23 strain. The virulome of *E. hormaechei* VDL/23 revealed the presence of several gene clusters classically associated with bacterial motility, environmental sensing and secretion. The VDL/23 strain harbours genes encoding flagellar biosynthesis and motility components, including the regulators *flhCD* and structural/motor elements (*fliA*, *fliC*, *fliG*, *fliI*, *fliM*, *flhA*, *motA* and *motB*). Additionally, genes related to chemotaxis regulation (*cheA*, *cheB*, *cheZ*, the methyl-accepting chemotaxis protein *tar/cheM* and the flagellar switch protein *fliY*) were identified. Furthermore, structural components of the Type VI secretion system (T6SS) were detected. Together, these systems are recognized as important fitness and virulence determinants in *Enterobacter* species. The detection of these genetic features in VDL/23 solely indicates a putative genetic basis for these mechanisms.^8^ Further functional validation is required to confirm their actual expression and their specific contribution to the strain’s pathogenicity and ecological fitness. Transconjugants exhibited resistance to both ceftazidime and meropenem, consistent with acquisition of the pVDL/23-2 plasmid. The transfer of *bla*_NDM-1_ and *bla*_CTX-M-15_ was confirmed by PCR. The conjugation frequency was 2.5 × 10^−5^ transconjugants per recipient. Bioinformatic analysis via MOB-suite identified a putative MOBF-type *oriT* region on pVDL/23-2; however, a comprehensive search failed to detect any canonical relaxase genes, type IV secretion system (T4SS) components, or integral *tra/trb* loci. Furthermore, the absence of a canonical *oriT* sequence was corroborated by oriTfinder. A noteworthy finding was the apparent discrepancy between the successful transfer of *bla*_NDM-1_ observed in conjugation experiments and the absence of identifiable complete conjugation machinery in the reconstructed pVDL/23-2 plasmid. Notably, the co-resident plasmid pVDL/23-1 harbours a Mate Pair Formation type F (MPFF) system, despite being classified as non-mobilizable. We hypothesize that pVDL/23-2 is transferred via mobilization *in trans* by exploiting the pVDL/23-1 conjugation machinery, potentially facilitated by a relaxase encoded elsewhere in the genome or by other co-resident MGEs.^20^ In summary, the NDM-1-producing *E. hormaechei* VDL/23 isolate belongs to the ST145 lineage, which has been increasingly associated with MDR and the dissemination of carbapenemase genes. To the best of our knowledge, this represents the first report of an NDM-1-producing *E. hormaechei* ST145 isolate in Italy. These findings underscore the importance of continuous genomic surveillance, infection prevention and control measures and antimicrobial stewardship to monitor and limit the dissemination of high-risk ECC clones in healthcare settings.

**Table 1.** Genomic characteristics of *E. hormaechei* VDL/23 strain

Genome	Size (bp)	%GC	Inc type	Antimicrobial resistance genes
Chromosome	4679906	55.25	NA	*bla*_ACT-90_ *; oqxB; oqxA; fosA*
pVDL/23-1	157232	53.34	IncFIB(pECLA); IncFII(pECLA)	none
pVDL/23-2	78963	51.99	IncFIA(HI1)	*bla*_CTX-M-15_ *; bla*_NDM-1_ *; bla*_OXA-1_ *; ble; aac(3)-IIa; aac(6’)-Ib; aac(6’)-Ib-cr5; dfrA14; catB3*
pVDL/23-3	2494	51.48	ND	none

NA, not applicable; ND, not determined
